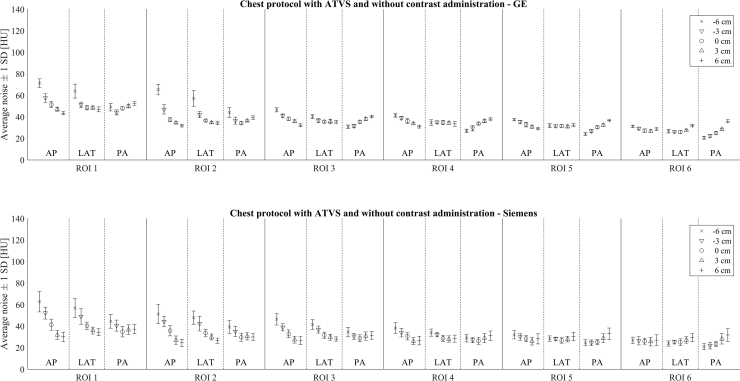# The effect of vertical centering and scout direction on automatic tube voltage selection in chest CT: a preliminary phantom study on two different CT equipments

**DOI:** 10.1016/j.ejro.2018.12.001

**Published:** 2018-12-17

**Authors:** Touko Kaasalainen, Teemu Mäkelä, Mika Kortesniemi

**Affiliations:** a HUS Medical Imaging Center, Helsinki University Central Hospital, Finland; b Department of Physics, University of Helsinki, Finland

**Keywords:** Automatic tube voltage selection, Image quality, Patient centering, Tube current modulation

## Abstract

**Purpose:**

To determine the effect of patient’s vertical off-centering and scout direction on the function of automatic tube voltage selection (ATVS) and tube current modulation (TCM) in chest computed tomography (CT).

**Methods:**

Chest phantom was scanned with Siemens and GE CT systems using three clinical chest CT protocols exploiting ATVS and a fixed 120 kVp chest protocol. The scans were performed at five vertical positions of the phantom (−6 to +6 cm from the scanner isocenter). The effects of scout direction (posterior-to-anterior, anterior-to-posterior, and lateral) and vertical off-centering on the function of ATVS and TCM were studied by examining changes in selected voltage, radiation dose (volume CT dose index, CTDI_vol_), and image noise and contrast.

**Results:**

Both scout direction and vertical off-centering affected ATVS. The effect differed between the vendors for the studied geometry, demonstrating differences in technical approaches. The greatest observed increase in CTDI_vol_ due to off-centering was 91%. Anterior-to-posterior scout produced highest doses at the uppermost table position, whereas posterior-to-anterior scout produced highest doses at the lowermost table position. Dose varied least using lateral scouts. Vertical off-centering impacted image noise and contrast due to the combined effect of ATVS, TCM, structural noise, and bowtie filters.

**Conclusions:**

Patient vertical off-centering and scout direction affected substantially the CTDI_vol_ and image quality in chest CT examinations. Vertical off-centering caused variation also in the selected tube voltage. The function of ATVS and TCM methods differ significantly between the CT vendors, resulting in differences in CTDI_vol_ and image noise characteristics.

## Highlights

• Patient vertical off-centering affects the function of ATVS and TCM.
• Patient vertical off-centering has impacts on radiation dose and image quality.
• CTDIvol was increased up to 91% with improper patient positioning.
• Scout direction affects the function of ATVS and TCM.
• The effect of vertical off-centering on ATVS and TCM differ between CT vendors.

## 1 Introduction

Medical radiation exposure to patients in diagnostics has increased mostly because of the growing use of computed tomography (CT) [[1], [2], [3]]. The increased use of CT in medical imaging has driven optimization efforts, including both technical and clinical approaches, to decrease radiation dose for the patients while maintaining the sufficient image quality for diagnosis. Technical optimization tools include, for example, tube current modulation (TCM) techniques, automatic tube voltage selection (ATVS), adaptive beam collimation, and iterative reconstruction methods [[4], [5], [6], [7], [8], [9], [10], [11], [12], [13], [14], [15], [16], [17], [18], [19]]. Despite the effective technical CT optimization tools, the role of user remains important to achieve optimal results both in terms of image quality and radiation dose. Several studies have previously shown remarkable effects of patient off-centering on patient radiation dose and image quality due to function of beam shaping filters and geometric magnification/minification resulted in the scout images (planning radiographs) [[20], [21], [22], [23], [24], [25], [26], [27], [28], [29], [30], [31], [32], [33], [34], [35], [36]]. The function of a bowtie filter is to allow maximum x-ray intensity to the thickest part of a patient and to reduce x-ray intensity in peripheral areas with less attenuation, thereby reducing x-ray scatter and radiation dose of surface tissues [37]. The optimal function of the bowtie filter and TCM techniques assume that the patient is centered on the scan isocenter.

Recently developed ATVS approaches aim to maintain a constant contrast-to-noise ratio between the examinations performed for different sized patients with as low radiation exposure as possible [14,19,38,39]. ATVS methods use scout images to determine the net attenuation profile of the patient. Based on the attenuation, ATVS algorithms determine the most dose-efficient combination of tube voltage and tube current settings to provide the needed image quality on a CT scan. Therefore, the ATVS presents a more general optimization tool which includes the TCM functionality to select the most optimal scan settings. While doing so, the ATVS also accounts for the examination type (e.g. CT angiography, contrast-enhanced scan, soft tissue or bone without contrast administration) in order to reach the optimal clinical image quality considering the availability and level of iodine contrast enhancement [19,38].

The impact of off-centering and scout direction on TCM have previously been extensively studied [[20], [21], [22], [23], [24], [25], [26], [27], [28], [29],[31], [32], [33], [34], [35], [36]]. However, the effect of off-centering on ATVS have only been studied for dose and not for image quality [30]. The effect of scout direction on ATVS has not been investigated. ATVS and TCM methods are strongly interconnected and their technical implementations vary between manufacturers. Therefore, it is necessary to study the impacts of these optimization tools in a comprehensive manner with clinical scan protocols. The aim of the current study was to determine the combined effect of the scout direction and patient’s vertical off-centering on ATVS and related TCM in chest CT examinations. Both radiation dose and image quality in these scans were investigated. The measurements with an anthropomorphic chest phantom were performed using two CT systems from different vendors.

## 2 Material and methods

### 2.1 Phantom measurements

**Fig. 1:**
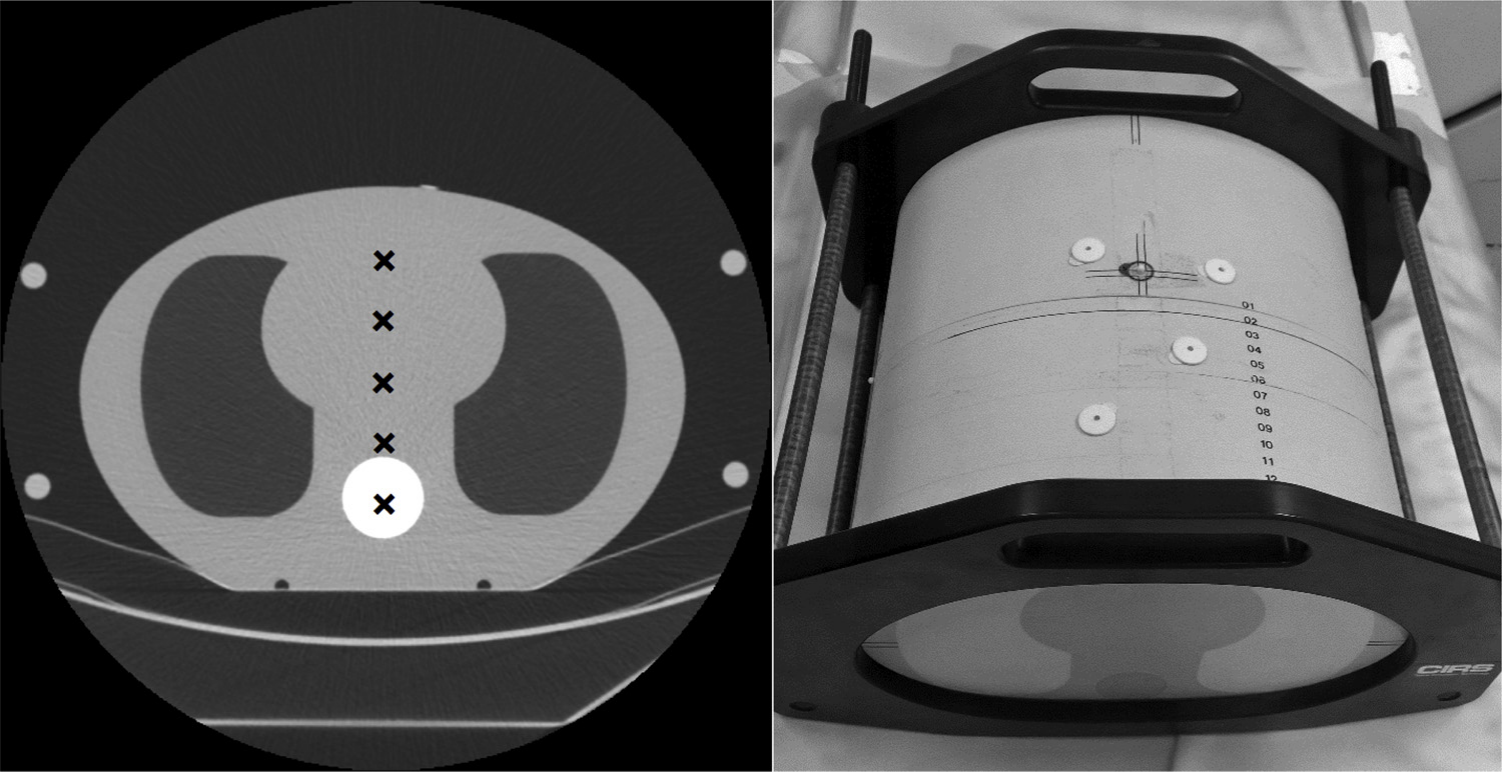
A 0.6 mm thick axial CT image (left) and a photograph (right) of the chest phantom. The phantom represented an average human torso in proportion, density, and two dimensional structures, and was constructed of three specific epoxy materials simulating lung, soft, and bone tissues. Black crosses on the left indicate the scan isocenter locations at the five studied vertical levels. These (from the bottom to the top) are referred in the article as: +6 cm (i.e. phantom is positioned too high), +3 cm, 0 cm, -3 cm, and -6 cm (i.e. phantom is positioned too low).

An anthropomorphic chest phantom (IMRT Thorax Phantom Model 002LFC, CIRS, Norfolk, USA) was scanned in a supine position on Siemens SOMATOM Definition Flash (Siemens Healthcare, Erlangen, Germany) and GE Revolution HD (GE Healthcare, Milwaukee, Wisconsin, USA) CT systems using clinical protocols for chest CT examinations. The four scanning protocols were 1) a chest CT protocol with ATVS and with contrast administration, 2) a chest CT protocol with ATVS and without contrast administration, 3) a chest CT protocol for pulmonary embolism with ATVS, and 4) a routine chest CT scan protocol with a fixed 120 kVp tube voltage. All protocols utilized TCM and were imaged without contrast agent. The scanning parameters and settings of the protocols are presented in Table 1. The phantom had an elliptical cross section and approximated human torso in proportion, density, and two-dimensional structure (Fig. 1). The phantom dimensions were 30 cm x 20 cm, and consisted of axial slabs of proprietary epoxy materials. According to the manufacturer, linear attenuations of the simulated tissues in the phantom were within 1% of the actual attenuation for water and bone, and within 3% for lung in the diagnostic X-ray energy range. The structure of the phantom did not vary in z-direction. The scan length was adjusted to 27.4 cm, leaving the phantom support parts out of the scan range. Large (“body”) bowtie filters were used for the scans.

**Table 1:** Scan protocols and used settings in the exposures (Siemens SOMATOM Definition Flash / GE Revolution HD).

Protocol	Detector configuration	Pitch	Rotation time	Quality reference mAs / Noise Index	Reference kVp and selection of examination type in ATVS
Chest CT scan for pulmonary embolism, ATVS	128 x 0.6 mm / 64 x 0.625 mm	1.2 / 1.375	0.5 s / 0.4 s	200 mAs / 50.5	100 kVp / slider position 9 (angiography) / CTA
Chest CT scan with contrast administration, ATVS	128 x 0.6 mm / 64 x 0.625 mm	1.2 / 1.375	0.5 s / 0.4 s	80 mAs / 50.5	120 kVp / slider position 7 (parenchymal) / Soft tissue, contrast-enhanced
Chest CT scan without contrast administration, ATVS	128 x 0.6 mm / 64 x 0.625 mm	1.2 / 1.375	0.5 s / 0.4 s	80 mAs / 50.5	120 kVp / slider position 3 (non-contrast) / Soft tissue, non-contrast
Chest CT scan with a fixed 120 kVp	128 x 0.6 mm / 64 x 0.625 mm	1.2 / 1.375	0.5 s / 0.4 s	80 mAs / 50.5	Fixed 120 kVp

CT = Computed tomography, ATVS = Automatic tube voltage selection, CTA = Computed tomography angiography.

The phantom was scanned in a helical mode at five different vertical levels of the patient table (from 6 cm below to 6 cm above the scan isocenter in 3 cm steps, see Fig. 1). The reference height position (0-level) was set using the lasers and landmarks on the phantom. To evaluate the effect of scout direction on ATVS and TCM, the phantom was scanned three times with each protocol and table height combination: using anterior-to-posterior (AP), posterior-to-anterior (PA), and lateral (LAT) scouts. For each scanning protocol and scout direction, the tube voltages selected by ATVS and the volume CT dose index (CTDI_vol_) values from the scanner console were recorded. Additionally, the apparent phantom size, measured as the projected width from the scout image, was determined in each vertical height position. Therefore, the apparent phantom size changed according to projected width magnification in the scout image.

### 2.2 Image analysis

The relative changes in image quality was evaluated from 0.75 mm (Siemens) and 0.625 mm (GE) thick axial reconstructions (512 × 512 pixels) of the phantom. The scans were reconstructed with clinically used reconstruction kernels/filters: pulmonary embolism scans were reconstructed with i26f kernel, whereas i31f kernel was used for other protocols on the Siemens CT system, and a standard reconstruction filter (“STND”) was used for all datasets on the GE CT system. Iterative reconstruction was used in all reconstructions: Safire level 2 for Siemens, and 40% ASIR-filtered back projection blending for GE. The axial display field of view (DFOV) was adjusted to 38 cm resulting in a pixel size of 0.742 × 0.742 mm^2^ in all the images.

**Fig. 2:**
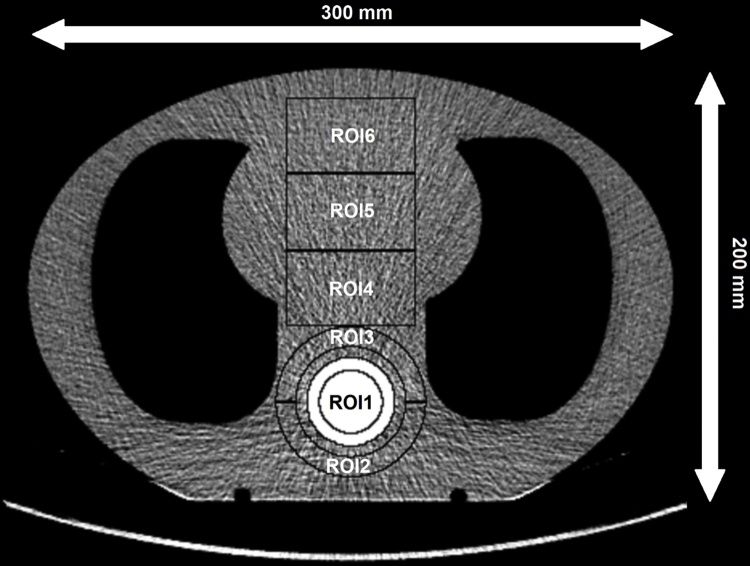
Region-of-interest (ROI) placement. The ROIs were defined to represent homogeneous soft-tissue areas in the relevant antero-posterior range in the phantom, and inside and around the stronger attenuation of the spine region. The ROI areas were: 700 mm^2^ for ROI 1, 900 mm^2^ for ROIs 2–3, and 2100 mm^2^ for ROIs.4–6.

The image noise was estimated by calculating CT number standard deviations (SDs) in six regions-of-interest (ROIs) shown in Fig. 2. ROI 1 was placed inside the spine and ROIs 2–6 were placed in the soft-tissue equivalent material. The SDs were calculated for 10 slices with 10 mm intervals (i.e. slabs 1–10 in Fig. 1B comprising a total z-direction coverage of 10 cm) to avoid the small gaps between phantom slabs. For each ROI, the mean of the ten SDs was reported as the noise value and the SD (of the 10 SDs) as the noise error.

Additionally, the effect of vertical centering was visualized for the fixed 120-kVp protocol. Absolute differences in CT numbers and relative noise differences between the off-centered and properly positioned phantom images (used as a reference) were calculated. The absolute CT numbers were calculated as the voxel-wise means and the noise maps as the voxel-wise SDs over the aforementioned 10 slices.

## 3 Results

### 3.1 Apparent width, radiation dose, and the function of ATVS

**Fig. 3:**
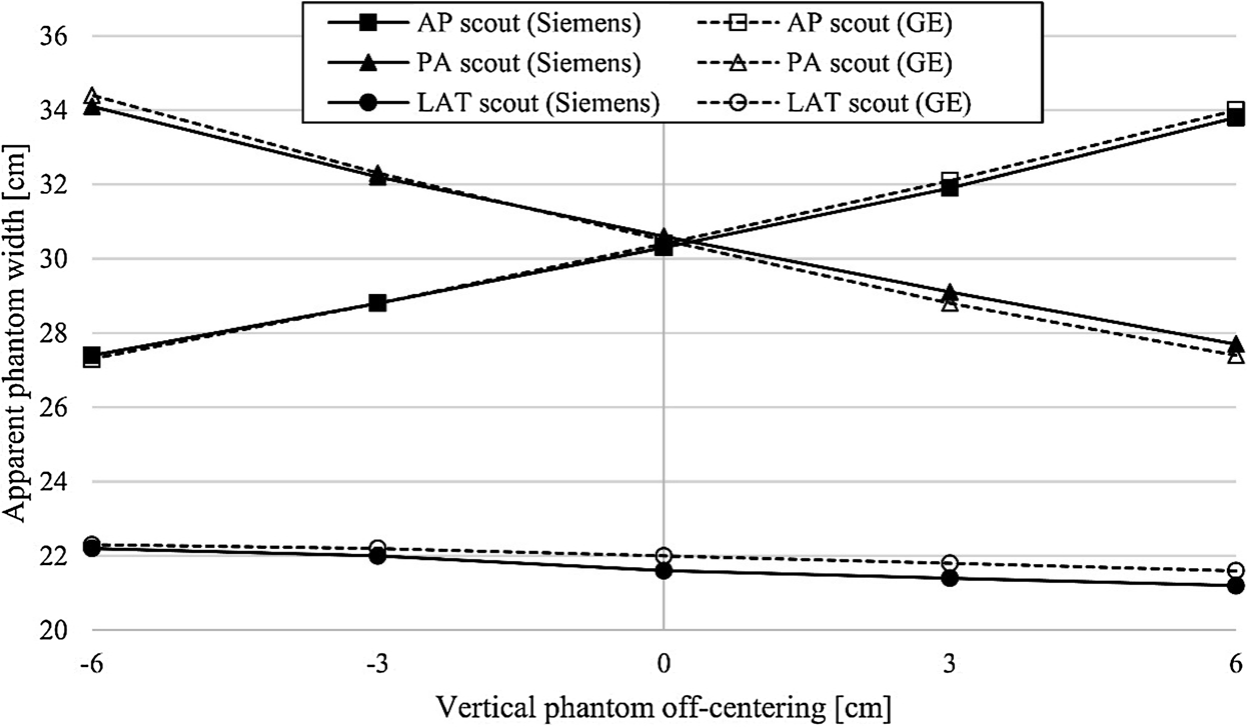
The apparent phantom widths measured from the scout images at different table heights. The effect of vertical positioning on the projected image magnification and minification was pronounced in the AP and PA scout directions (true phantom right-to-left length was 30 cm), whereas only a minimal effect was seen using the lateral scout (true length in AP direction was 20 cm).

Fig. 3 shows the effect of phantom’s vertical off-centering on the projected size of the phantom in the scout images. In the Siemens CT system, the measured phantom widths varied due to geometric magnification and minification between 27.4 cm–33.8 cm, 27.7 cm–34.1 cm, and 21.2 cm–22.2 cm in AP, PA, and lateral scout images, respectively. Similarly in the GE CT system, the corresponding widths were 27.3 cm–34.0 cm, 27.4 cm–34.4 cm, and 21.6 cm–22.3 cm in AP, PA, and lateral scout images, respectively.

Table 2, Table 3, Table 4, Table 5 show the effects of phantom’s vertical off-centering on the function of ATVS and the CTDI_vol_ values in the four investigated protocols. There were differences in the behavior of ATVS techniques between the Siemens and GE CT systems for the studied geometry. The Siemens’ ATVS technique selected systematically higher tube voltages after AP and PA scouts compared to lateral scout, whereas the GE’s ATVS technique tended to select more likely a lower tube voltage after AP and PA scouts compared to lateral scout. Moreover, GE’s ATVS technique presented less voltage variance compared to the Siemens’ ATVS approach. In the GE CT system, the tube voltage varied only in the pulmonary embolism scans, whereas in the Siemens CT system, variance was seen in all the protocols utilizing ATVS. In pulmonary embolism scan, the magnification or minification of the spine caused changes in the tube voltage selection on the Siemens system in AP scout (off-centering -3 cm and -6 cm) compared to PA scout (off-centering +3 cm and +6 cm). ATVS chose 100 kVp tube voltage in the former, and 120 kVp in the latter cases.

**Table 2:** Volume CT dose indices (CTDI_vol_) and tube voltages at different phantom’s vertical positions in the pulmonary embolism chest CT protocols using ATVS. The tube voltages changed compared to the reference table height are bolded.

Patient vertical position (cm)	AP scout (Siemens)		AP scout (GE)		PA scout (Siemens)		PA scout (GE)		LAT scout (Siemens)		LAT scout (GE)	
	CTDI (mGy)	Tube voltage (kVp)	CTDI (mGy)	Tube voltage (kVp)	CTDI (mGy)	Tube voltage (kVp)	CTDI (mGy)	Tube voltage (kVp)	CTDI (mGy)	Tube voltage (kVp)	CTDI (mGy)	Tube voltage (kVp)
+6	6.14	120	3.17	**100**	5.63	120	1.30	80	2.48	80	2.55	100
+3	5.89	120	2.07	80	6.22	120	1.51	80	2.43	80	2.58	100
0	5.27	120	1.74	80	6.67	120	1.78	80	2.35	80	2.56	100
−3	3.18	**100**	1.48	80	6.96	120	2.25	80	2.37	80	2.49	100
−6	3.00	**100**	1.28	80	7.10	120	3.40	**100**	2.40	80	1.98	**80**

CTDI = Computed tomography dose index, AP = Anterior-to-posterior, PA = Posterior-to-anterior, LAT = Lateral.

**Table 3:** Volume CT dose indices (CTDI_vol_) and tube voltages at different phantom’s vertical positions in routine chest CT protocols with contrast administration using ATVS. The tube voltages changed compared to the reference table height are bolded.

Patient vertical position (cm)	AP scout (Siemens)		AP scout (GE)		PA scout (Siemens)		PA scout (GE)		LAT scout (Siemens)		LAT scout (GE)	
	CTDI (mGy)	Tube voltage (kVp)	CTDI (mGy)	Tube voltage (kVp)	CTDI (mGy)	Tube voltage (kVp)	CTDI (mGy)	Tube voltage (kVp)	CTDI (mGy)	Tube voltage (kVp)	CTDI (mGy)	Tube voltage (kVp)
+6	4.22	**120**	3.15	100	3.75	120	1.70	100	2.00	**80**	2.54	100
+3	3.89	**120**	2.60	100	4.20	120	1.94	100	1.95	**80**	2.53	100
0	2.61	100	2.17	100	4.51	120	2.28	100	1.91	100	2.51	100
−3	2.37	100	1.87	100	4.87	120	2.86	100	1.94	100	2.47	100
−6	2.20	100	1.63	100	5.21	120	3.43	100	1.96	**80**	2.41	100

CTDI = Computed tomography dose index, AP = Anterior-to-posterior, PA = Posterior-to-anterior, LAT = Lateral.

**Table 4:** Volume CT dose indices (CTDI_vol_) and tube voltages at different phantom’s vertical positions in routine chest CT protocols without contrast administration using ATVS. The tube voltages changed compared to the reference table height are bolded.

Patient vertical position (cm)	AP scout (Siemens)		AP scout (GE)		PA scout (Siemens)		PA scout (GE)		LAT scout (Siemens)		LAT scout (GE)	
	CTDI (mGy)	Tube voltage (kVp)	CTDI (mGy)	Tube voltage (kVp)	CTDI (mGy)	Tube voltage (kVp)	CTDI (mGy)	Tube voltage (kVp)	CTDI (mGy)	Tube voltage (kVp)	CTDI (mGy)	Tube voltage (kVp)
+6	4.42	**120**	3.72	120	3.48	**100**	2.00	120	2.61	100	2.99	120
+3	4.03	**120**	3.04	120	3.72	**100**	2.30	120	2.56	100	2.98	120
0	3.21	100	2.58	120	4.67	120	2.69	120	2.42	100	2.96	120
−3	2.85	100	2.21	120	4.87	120	3.33	120	2.33	100	2.91	120
−6	2.68	100	1.93	120	5.41	120	4.00	120	2.37	100	2.82	120

CTDI = Computed tomography dose index, AP = Anterior-to-posterior, PA = Posterior-to-anterior, LAT = Lateral.

**Table 5:** Volume CT dose indices (CTDI_vol_) and tube voltages at different phantom’s vertical positions in routine chest CT protocols without contrast administration using a fixed 120 kVp tube voltage.

Patient vertical position (cm)	AP scout (Siemens)		AP scout (GE)		PA scout (Siemens)		PA scout (GE)		LAT scout (Siemens)		LAT scout (GE)	
	CTDI (mGy)	Tube voltage (kVp)	CTDI (mGy)	Tube voltage (kVp)	CTDI (mGy)	Tube voltage (kVp)	CTDI (mGy)	Tube voltage (kVp)	CTDI (mGy)	Tube voltage (kVp)	CTDI (mGy)	Tube voltage (kVp)
+6	4.22	120	3.70	120	3.83	120	1.99	120	2.62	120	2.96	120
+3	3.89	120	3.06	120	4.22	120	2.29	120	2.56	120	2.98	120
0	3.58	120	2.57	120	4.62	120	2.68	120	2.48	120	3.00	120
−3	3.27	120	2.21	120	4.79	120	3.33	120	2.51	120	2.91	120
−6	2.98	120	1.93	120	5.24	120	4.03	120	2.53	120	2.82	120

CTDI = Computed tomography dose index, AP = Anterior-to-posterior, PA = Posterior-to-anterior, LAT = Lateral.

As could be expected based on the projected magnification of the scout image, the CTDI_vol_ values were the greatest with PA scout when the phantom was centered at the lowest table height position, and the lowest when the phantom was at the highest table height position (as a combined effect of ATVS and TCM functions, respectively). Conversely, in the case of AP scout, the CTDI_vol_ values were the greatest when aligning the phantom at the highest table position and lowest when the phantom was centered at the lowest table position. When the lateral scout was used for ATVS and TCM, the changes in CTDI_vol_ values were more subtle than when using AP or PA scouts.

**Fig. 4:**
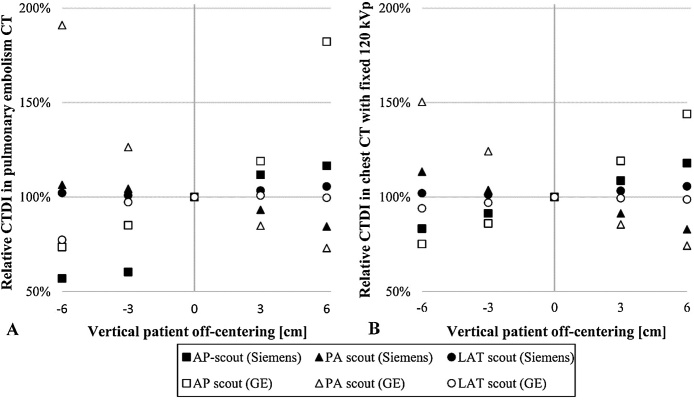
Relative CTDI_vol_ values as a function of phantom’s vertical position in chest CT scans for pulmonary embolism using ATVS (A) and routine chest CT scans with the fixed 120 kVp tube voltage (B).

Fig. 4 shows the relative doses at each table height position and scout direction compared to reference table positions in pulmonary embolism protocol utilizing ATVS (A), and routine chest CT protocol with fixed 120 kVp tube voltage (B). Notable increase in CTDI_vol_ was observed whenever the tube voltage was increased in pulmonary embolism scans. Similar steps in CTDI_vol_ values were also seen in CT scans performed with the non-contrast and contrast-enhanced chest CT scan protocols using ATVS. The highest change in relative dose (91%) was observed with the GE scanner when performing a CT scan for pulmonary embolism after PA scout and when the phantom was set 6 cm below the scanner isocenter. This was related to the larger magnification of the spine structure with higher attenuation compared to soft-tissue and lung areas, thereby contributing more to the automatically adjusted dose level. In the scans performed with the fixed 120 kVp tube voltage, the highest change in relative dose (50%) was seen in the GE system after PA scout and when the phantom was positioned 6 cm below the scan isocenter, whereas the largest change with Siemens system was 18%. Overall, the CTDI_vol_ alterations were higher with the GE scanner than the Siemens system, probably due to the differences in beam shaping filters of the scanners and differences in the TCM systems. GE noise index model involves stronger variability in tube currents pursuing to constant noise statistics in the images, whereas Siemens CARE Dose 4D has smoother response according to patient attenuation [31].

### 3.2 Image analysis

**Fig. 5:**
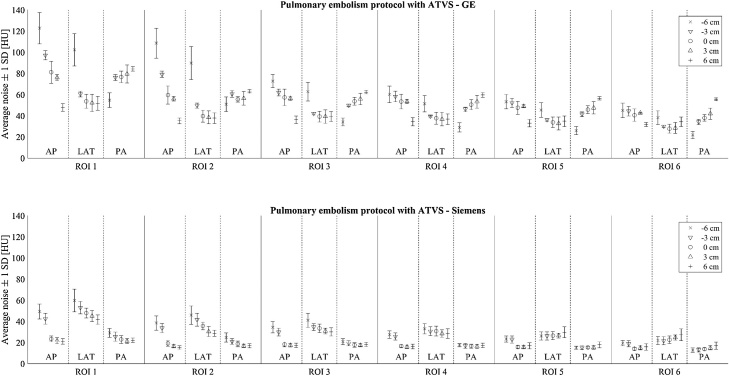
Average image noise values (±1 SD) over ten slices in different phantom regions using pulmonary embolism protocols with ATVS. The upper and lower images show the corresponding values for GE and Siemens systems, respectively. Noise values are calculated for six regions-of-interest (ROI 1 is spine, ROIs 2–6 are in soft tissue, see Fig. 2) using three scout directions (AP, LAT, PA) and at five vertical table positions (phantom center -6 to +6 cm from the CT scanner isocenter).

**Fig. 6:**
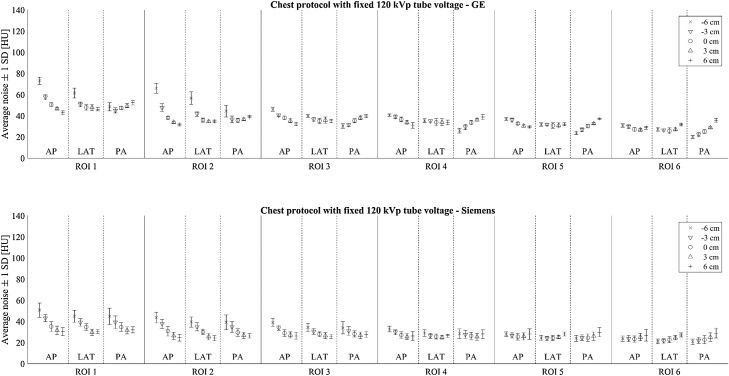
Average image noise values (±1 SD) over ten slices in different phantom regions using chest CT protocol with fixed 120 kVp tube voltage. The upper and lower images show the corresponding values for GE and Siemens systems, respectively. Noise values are calculated for six regions-of-interest (ROI 1 is spine, ROIs 2–6 are in soft tissue, see Fig. 2) using three scout directions (AP, LAT, PA) and at five vertical table positions (phantom center -6 to +6 cm from the CT scanner isocenter).

Fig. 5, Fig. 6 show the image noises in different phantom regions when using pulmonary embolism protocols with ATVS and routine chest CT protocols with fixed 120 kVp tube voltage, respectively. The image noise values measured in the chest CT protocols with and without the defined contrast administration, both using ATVS, are given in the Supplementary materials Figs. A.1 and A.2, respectively. Clear steps in the image noise can be seen in the pulmonary embolism scans (Fig. 5) whenever the tube voltage was changed. Lower tube voltages yielded higher image noise with both scanners. Image noise was higher in bone tissue (ROI 1) compared to soft tissues (ROIs 2–6). Moreover, the vertical centering had greater impact on the noise in the peripheral regions compared to the central region (ROIs 3–4). Beam hardening and streak artefacts contributed to additional local and structural noise component which caused the overall image noise to vary more in ROI 2 than in ROI 3. This can be seen in wider error bars in the Fig. 5, Fig. 6. Noise behavior differences between the scanners, especially in the posterior parts of the phantom, are most probably caused by bowtie filter and TCM method differences. Siemens is using a real-time TCM in addition to scout-based TCM while GE system is only using the last scout image for TCM. Additionally, Siemens system allows higher image noise levels for obese patients and lower noise levels for small patients. GE system, on the other hand, tries to deliver the same noise level, regardless of the patient body size.

**Fig. 7:**
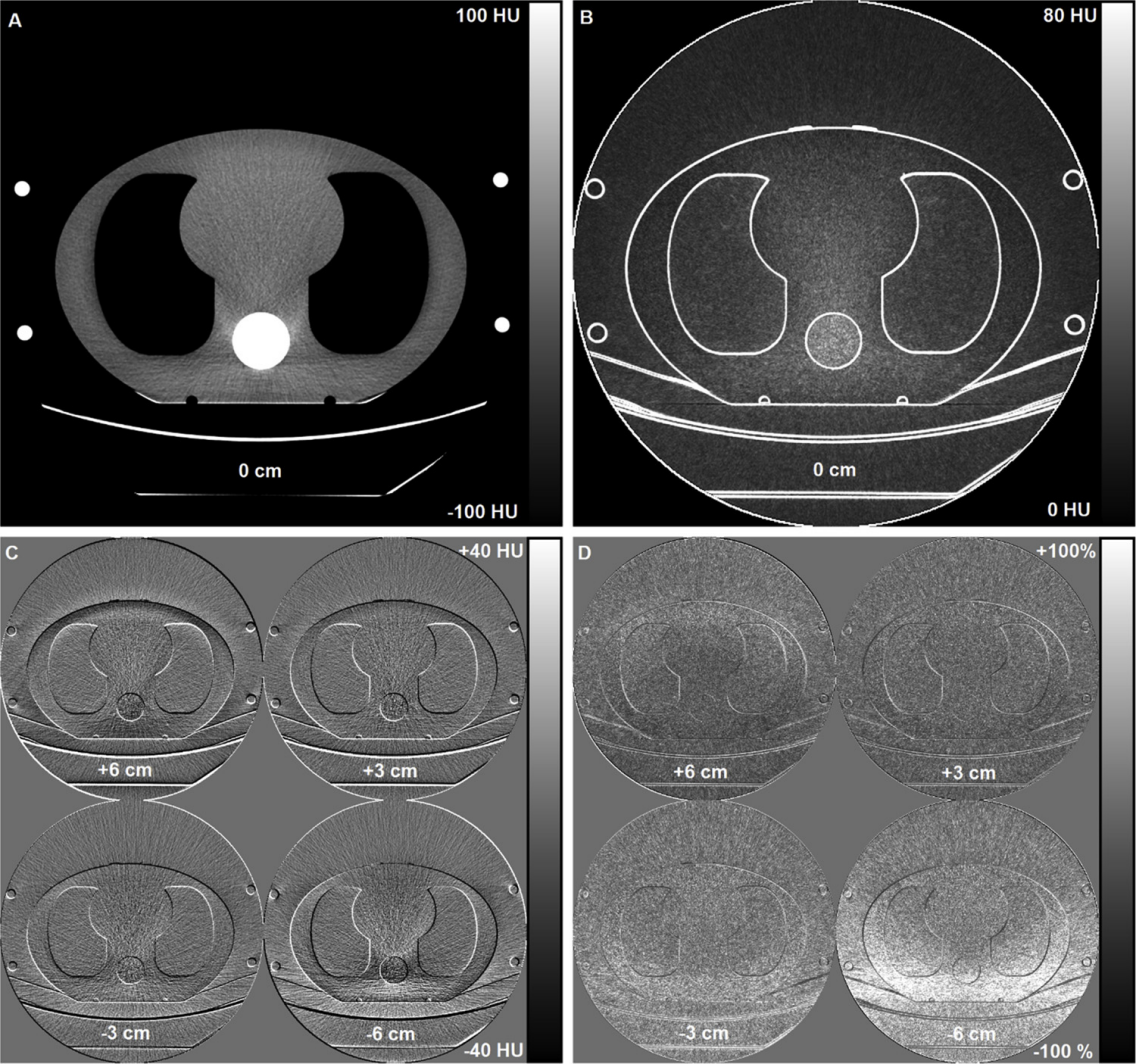
Axial image data from the GE chest CT scan with a fixed 120 kVp tube voltage after an AP scout. The reference axial CT image with mean CT numbers (A) and corresponding 1SD noise map (B) were calculated from ten slices comprising a total z-direction volume coverage of 10 cm. Absolute CT number differences (C) and relative noise difference maps (D) at table heights +6, +3, -3, and -6 cm were calculated relative to the reference centering (0 cm).

**Fig. 8:**
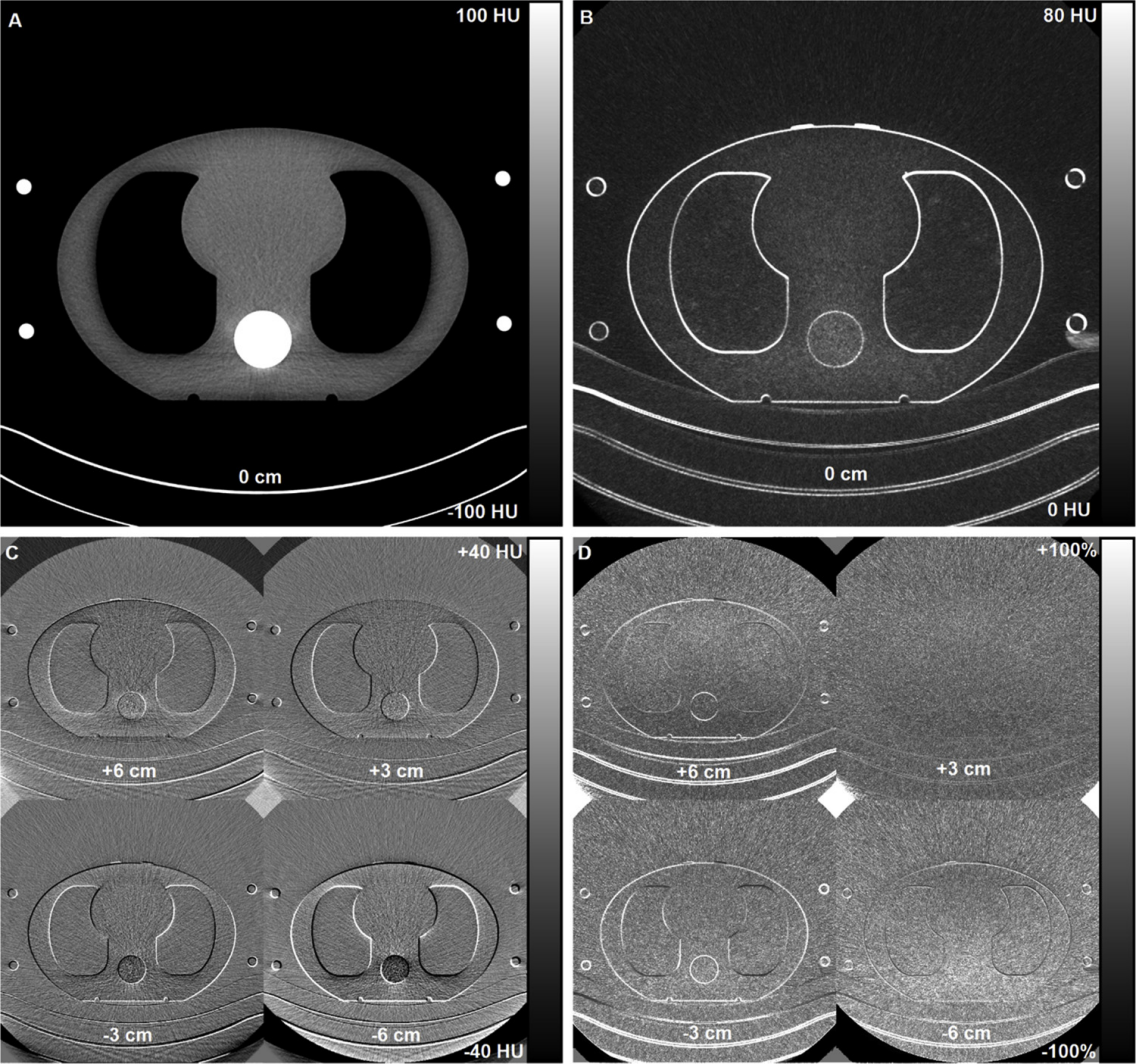
Axial image data from the Siemens chest CT scan with a fixed 120 kVp tube voltage after an AP scout. The reference axial CT image with mean CT numbers (A) and corresponding 1SD noise map (B) were calculated from ten slices comprising a total z-direction volume coverage of 10 cm. Absolute CT number differences (C) and relative noise difference maps (D) at table heights +6, +3, -3, and -6 cm were calculated relative to the reference centering (0 cm).

Figs. 7 and 8 show the difference maps for the GE and Siemens CT scanners, respectively. As the noise difference maps show, with the fixed 120-kVp protocol and AP scout direction, image noise on the posterior side was the greatest when the phantom was positioned 6 cm below the isocenter, and the smallest when the phantom was on the highest table position. On the contrary, image noise on the anterior side was the greatest when the phantom was positioned 6 cm above the isocenter of the CT scanners, and the smallest with the lowest table position. The image noise changes in the GE scanner were higher than with the Siemens scanner for the studied geometry. The PA and LAT scout directions resulted in similar noise behavior as the AP scout direction.

Phantom’s vertical off-centering affected not only the image noise but also the image contrast. Fig. 7, Fig. 8 show that the relative CT numbers altered the most in bone tissue but also to some extent in the soft and lung tissues when the phantom was vertically off-centered. There were visible differences between the vendors in the studied geometry. For example, with +6 cm centering, the CT numbers in the phantom’s anterior side deviated more on the GE system compared to the Siemens system, whereas the overall variations in the bone CT numbers were smaller in the GE system.

## 4 Discussion

Several studies have shown that patient off-centering is a common and serious problem in CT with detrimental effects on patient dose and image quality [[20], [21], [22], [23], [24], [25], [26], [27], [28], [29], [30], [31], [32], [33], [34], [35], [36]]. According to these publications, patients are typically positioned 1.7 cm–3.5 cm below the scan isocenter. The previous patient positioning studies have mainly investigated the function of TCM and only in a single limited study has the effect of ATVS on radiation dose been investigated [30]. No study to date has systematically assessed the effect of patient off-centering on the function of ATVS with different scout directions and different CT vendors.

Due to divergent x-ray fan beam geometry with relatively short focus-to-patient distance in CT, patients are projected larger or smaller to the detector surface depending of the patient alignment in the focus-detector axis (off-centering). This has an effect not only on the selected tube current in the TCM but also the function of ATVS because both techniques utilize scout images in the calculation of the patient’s net attenuation. Body regions with higher attenuation or patients with larger cross-sectional size are then scanned with using higher tube currents or tube voltages compared to the body regions with lower x-ray attenuation or patients with smaller cross-sectional size. As Fig. 3 showed, a vertical off-centering of the phantom causes geometric magnification or minification in the acquired scout images, especially in the AP or PA directions. Similarly, lateral off-centering, if present, would cause geometric magnification or minification for the lateral scout images.

The results of the current phantom study revealed clear differences in CTDI_vol_ values and image noise levels when the phantom was scanned in off-centered vertical positions compared to properly positioned scans on the scan isocenter. There were also differences observed between the manufacturers in the function of ATVS. The GE’s ATVS worked with less voltage variance than the Siemens’ ATVS technique in the studied geometry. The Siemens system tended to select higher tube voltage after the AP and PA scouts compared to the lateral scout, whereas in the GE CT scanner, either higher or the same tube voltage was selected after the lateral scout compared to the scans performed after AP or PA scouts. The difference was most evident in the pulmonary embolism scans. In practice, if ATVS behaves inconsistently or systematically results in suboptimal noise levels, the tube voltage range, patient centering practices, or protocol scout directions might have to be reconsidered. However, if the scout direction is changed, the noise indices or quality reference mAs levels may have to be readjusted to assure consistent image quality according to indication.

The scanner reported radiation dose, measured in CTDI_vol_, increased substantially in the highest or lowest table positions compared to the reference table height. Furthermore, the dose absorbed by sensitive surface tissues such as the breast and thyroid gland will also increase if the patient in supine position is positioned too low and the PA scout direction is used for the TCM and ATVS. These anterior sensitive tissues are then located closer to the scan isocenter and thereby projected on the thinnest and least attenuating part of the bowtie filter during the scan rotation [25,32].

Image analysis results of different ROIs (see e.g. Fig. 6 for the fixed 120 kVp scans) demonstrated distinct noise trends. Higher observed noise values of the posterior ROIs 1–3 while the phantom was in the lower positions (centered 6 cm and 3 cm below scan isocenter) were due to a lower photon flux on these locations and being farther away from the scan isocenter. In this respect, the lower photon flux is partly due to the bowtie filter being thinner at the center, allowing more x-rays through, and thicker at the edge, attenuating more x-rays in these parts of the fan beam. The same effect of higher noise values was seen on the anterior ROIs (ROIs 5 and 6) while the phantom was in the higher positions (centered 6 cm and 3 cm above scan isocenter). The effect depends also on the automatic TCM and scout direction. There was a trend of lower noise levels with increasing centering heights with AP scout, and vice versa with the PA scout, with the GE scanner and most clearly seen in the central ROI 4. This was mostly due to the scout direction-based minification or magnification of the phantom structures, especially the spine, which influence accordingly to the TCM. Therefore, the phantom attenuation appears larger in the PA scout when the phantom is deviated into lower position and closer to the x-ray tube, causing TCM to use higher tube currents and leading to decreased noise levels in the CT images, respectively. This effect was not seen the same way in the Siemens scanner, probably due to a different TCM technique which utilizes scout image in longitudinal modulation and on-line projection data in angular modulation during the helical CT scan. Therefore, the significance of the scout direction on noise levels was not as pronounced with the Siemens system. Additionally, the effect of scout direction on noise levels was not as pronounced with the PA scout for posterior ROIs, and vice versa with the AP scout direction for anterior ROIs. This was due to the combined effect of automatic TCM, ROI location with respect to the scan, and bowtie differences (Siemens’ bowtie filter being flatter than in GE) between the two vendors.

Patient centering affected not only the radiation dose and image noise, but also image contrast. A change in tube voltage affects the x-ray spectra, and thus the x-ray attenuation-based CT-numbers. With lower photon energies, photoelectric effect is more prominent and less Compton scattering infers the detected signal, which is a clear benefit especially in CT angiography and contrast-enhanced CT imaging where iodine k-edge plays an important role in the contrast formation. Thus, as a result of using lower tube voltages, higher contrast between the iodine and soft tissue can be achieved [40]. The change in image contrast is also affected by the bowtie filters of the CT systems which modify the x-ray spectra assuming the patient as a cylindrical attenuation target for compensation. With patient off-centering, the bowtie compensating effects which assume isocenter positioning are misplaced with regards to the actual off-centered patient attenuation. Therefore, there will also be corresponding deviations in the reconstructed image noise and contrast (based on the relative x-ray attenuation) distribution across the axial scan plane direction [25,33]. In addition to the effects caused by vendor-specific TCM function and bowtie geometry, the observed image quality and radiation dose differences between the vendors may also be related to the slight differences in the focus-to-isocenter distances between the two scanner models (Siemens 595 mm and GE 541 mm). This also supports the flatter contrast and noise difference maps observed in the Siemens images.

The results of this study are consistent with the findings of Filev et al. [30] who found that the function of ATVS and TCM depend on patient centering. They observed that geometric magnification on PA scout images might result in higher tube voltages and tube current values, and thus also higher radiation doses if ATVS was used. However, they only used a Siemens CT system and PA scout direction whereas the current study included a GE CT system as well as AP and LAT scouts. Moreover, the results with the fixed 120 kVp tube voltage support the dosimetric and image noise findings of previous studies [[20], [21], [22], [23], [24], [25], [26], [27],^29^,[31], [32], [33],^35^]. When using the AP scout for TCM and positioning the phantom 6 cm above the isocenter, 18% and 44% increase in CTDI_vol_ values were observed in the Siemens and GE CT systems, respectively. The PA scout resulted in comparable dose increases (around 13% and 50%) when the phantom was centered 6 cm below the scan isocenter. Habibzadeh et al. [24] reported dose penalties of 13%, 33%, and 51% with 2 cm, 4 cm, and 6 cm vertical position errors for a phantom, respectively. Toth et al. [21] observed that off-centering the phantom by 3 cm and 6 cm increased the surface dose of a cylindrical 32 cm body CTDI phantom by 18% and 41% and the image noise by 6% and 22%, respectively. Kaasalainen et al. [25] found a 16% increase in the absorbed dose of breast tissue when a five-year-old pediatric phantom was scanned with the fixed mAs values and positioned 6 cm below the scan isocenter. Saltybaeva and Alkadhi [32] reported up to 38% increased thyroid dose when off-centering the anthropomorphic phantom by 5 cm. Moreover, Kaasalainen et al. [27] reported 38%, 21%, and 12% increased doses for the adult, five-year-old pediatric, and newborn anthropomorphic phantoms, respectively, when positioning the phantoms 6 cm below the isocenter and using PA scout for TCM in GE CT system.

The present study has certain limitations. Firstly, the study was performed using only one anthropomorphic chest phantom. For more extensive study, the effect of patient’s vertical off-centering should also be investigated on varying chest anatomy and with other anatomical body regions as well. Secondly, only two CT scanners from two vendors were studied, and thereby, the results may not be valid for other ATVS implementations. Thirdly, only CTDI_vol_ values were used to measure radiation doses, and further investigations to measure organ doses, for example, with MOSFET dosimeters and Monte Carlo simulations could be performed.

## 5 Conclusions

The scout direction and vertically off-centering the patient have complex and mixed effects on the ATVS and TCM operation, propagating further on the measured radiation dose and image quality. This emphasizes the importance of proper centering of the patient with the modern CT scanner models with new optimization tools. The greatest variation in the selected tube voltage by ATVS was seen in the pulmonary embolism scans. Furthermore, there were notable differences in the ATVS behavior between the CT vendors for the studied geometry. This emphasizes the importance of adequate in-depth knowledge of the users on the functionality of their scanner model and how optimization tools, centering and scan parameters affect both radiation dose and image quality.

## Declaration of interest

None.

## Confilict of interests

The authors do not have any competing interests to declare.

## Funding

This study was supported by the State Subsidy for University Hospitals in Finland and a research grant from the Radiological Society of Finland.

## Figures and Tables

**Figure:**
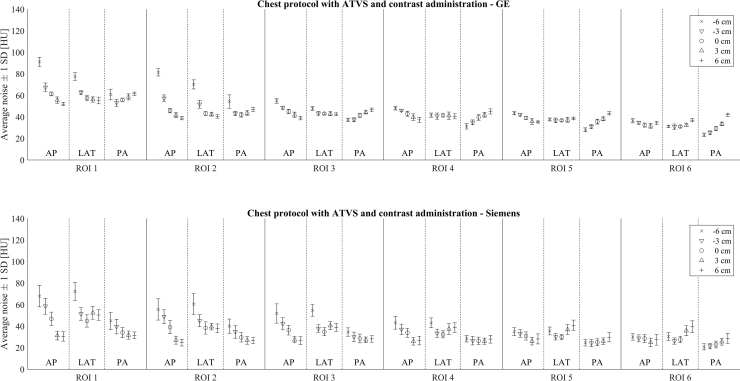


**Figure:**