# Supplementary material for: The effect of vertical centering and scout direction on automatic tube voltage selection in chest CT: a preliminary phantom study on two different CT equipments
Source: Eur J Radiol Open. 2018 Dec 17;6:24–32. doi: 10.1016/j.ejro.2018.12.001 (PMC6298908; doi:10.1016/j.ejro.2018.12.001)
Supplement: Supplementary file 1 [file mmc1.docx]

**Supplementary files:**

**Figure legends for the supplementary figures:**

**Fig. A.1.** Average image noise values (±1 SD) over ten slices in different phantom regions using contrast-enhanced chest CT protocol with ATVS. The upper and lower images show the corresponding values for GE and Siemens systems, respectively. Noise values are calculated for six regions-of-interest (ROI 1 is spine, ROIs 2-6 are in soft tissue, see Fig. 2) using three scout directions (AP, LAT, PA) and at five vertical table positions (phantom center -6 to +6 cm from the CT scanner isocenter).

**Fig. A.2.** Average image noise values (±1 SD) over ten slices in different phantom regions using non-contrast chest CT protocol with ATVS. The upper and lower images show the corresponding values for GE and Siemens systems, respectively. Noise values are calculated for six regions-of-interest (ROI 1 is spine, ROIs 2-6 are in soft tissue, see Fig. 2) using three scout directions (AP, LAT, PA) and at five vertical table positions (phantom center -6 to +6 cm from the CT scanner isocenter)
